# Dissemination of Pediatric Chronic Pain Research: Views from Patients, Caregivers, and Healthcare Professionals

**DOI:** 10.2147/JPR.S560855

**Published:** 2026-02-20

**Authors:** Jana Hochreuter, Emil Kane Nissen, Alice Prchal, Cosima Locher, Sabina C Heuss, Carina Rita Grätzer, Helen Koechlin

**Affiliations:** 1 Department of Psychosomatics and Psychiatry, University Children’s Hospital, University of Zurich, Zurich, Switzerland; 2 Department of Psychology, University of Zurich, Zurich, Switzerland; 3 Children’s Research Centre, University Children’s Hospital, University of Zurich, Zurich, Switzerland; 4 Faculty of Psychology, University of Basel, Basel, Switzerland; 5 Department of Consultation-Liaison Psychiatry and Psychosomatic Medicine, University Hospital Zurich, Zurich, Switzerland; 6 Institute for Competitiveness and Communication, University of Applied Sciences and Arts Northwestern Switzerland, Olten, Switzerland

**Keywords:** pediatric chronic pain, information dissemination, adolescents, caregivers, healthcare professionals

## Abstract

**Introduction:**

Around 25% of children and adolescents report chronic pain, which is often associated with a range of negative consequences. Targeted dissemination of research findings is crucial to inform patients, families, and healthcare professionals about chronic pain and its treatment. To do so, a key step is to identify the knowledge needs of the targeted audience, and to determine how to best reach them.

**Objective:**

We investigated satisfaction with current knowledge transfer concerning pediatric chronic pain and its treatment amongst adolescents with chronic pain, their parents, and healthcare professionals.

**Methods:**

Focus groups and semi-structured interviews with adolescents with chronic pain, parents of children with chronic pain, and healthcare professionals were carried out. The discussions and interviews were analyzed applying structural content analysis.

**Results:**

Three adolescents with chronic pain, four caregivers and twelve healthcare professionals from Switzerland participated in the study. Patients and caregivers highlighted barriers related to social, medical, organizational, and information-specific areas, and expressed a strong desire for more tailored and accessible support. Participants emphasized the importance of recipient-specific dissemination strategies that address both informational preferences and contextual realities. Healthcare professionals, while well-networked within Switzerland, emphasized the importance of access to pediatric research data, specifically from Swiss populations.

**Conclusion:**

Dissemination of scientific results on pediatric chronic pain to those who need it remains inadequate. The findings highlight the urgent need for pain education resources targeted to the different audiences, ensuring that affected families and healthcare providers are equipped with the information they need.

## Plain Language Summary

Chronic pain affects about 1 in 4 children and teenagers, yet many young people with chronic pain, their families, and healthcare professionals struggle to find reliable, easy-to-understand information about it. We wanted to understand what information people currently use, what information they wish they had, and what barriers they face when searching for it.

We spoke with three teenagers with chronic pain, four parents of children with chronic pain, and twelve healthcare professionals in Switzerland who work with young patients with pain. Through group discussions and individual interviews, we learned about their experiences and needs.

Teenagers and parents told us they feel overwhelmed by online information, and struggle to know which sources to trust. They wished doctors had more time to explain things clearly and provide simple summaries to take home. Many felt healthcare professionals did not take their pain seriously or did not know enough about chronic pain in young people. They also said society does not understand that chronic pain is real and deeply affects youth’s daily lives.

Healthcare professionals explained that they rely on scientific research and colleagues for information, but emphasized there is not enough research on children and teenagers with chronic pain in Switzerland. They want more treatment guidelines and better training.

These findings show that everyone affected by pediatric chronic pain needs better access to clear, trustworthy information tailored to their needs. Creating centralized resources, improving medical training, and raising public awareness about chronic pain in young people are essential steps forward.

## Introduction

The prevalence of chronic pain in children and adolescents has increased over the past years, with around 25% of children and adolescents reporting pain that lasts or recurs for three or more months.^1^,^2^ Chronic pain in childhood and adolescence tends to persist into adulthood,^3–5^ with manifold negative consequences in physical, psychological, social, and school domains.^6–8^ Moreover, recent research has found chronic pain in adolescence to predict first lifetime onset suicidality.^9^ The biopsychosocial model of chronic pain is well-established and helps to explain how biological, psychological, and social factors interact dynamically, and contribute to the pain experience and pain chronification.^10–12^

In clinical practice, chronic pain is often misunderstood or incompletely assessed: A recent review showed that while the biopsychosocial model of chronic pain is well-known within the healthcare system, certain key areas (eg, mobility, quality of sleep, school function) are not consistently included in chronic pain assessments.^13^ Pediatricians, who are usually the first-line contact for pain in children and adolescents, are often convinced that pediatric chronic pain is mostly explained by psychosocial factors, potentially resulting in limited or insufficient pain education of patients and families.^14^ In a study conducted in Switzerland, only 20.5% of the pediatricians surveyed (total N=337) felt confident about treating chronic pain, and 78.5% of the sample reported that they had not received any specific training in the diagnosis and treatment of pediatric chronic pain.^15^ This indicates that healthcare professionals often receive insufficient training in treating children with chronic pain.^13^,^16^

To address these gaps, integrative pain education should be prioritized and systematically embedded within medical training curricula. This would support the development of a shared educational foundation for physicians and promote effective management of chronic pain, including the application of integrative treatment approaches.^17^,^18^ Furthermore, there is a demand for such training among resident physicians, who have expressed a clear interest in learning more about integrative health strategies for pain management.^19^,^20^ Implementing a multidisciplinary pain education curriculum for pediatric residents might be effective, as it can influence how residents conceptualize, approach, and manage pain in clinical settings.^19^

Healthcare professionals, but also patients and their families, need access to new scientific findings in order to make informed decisions about the diagnostic process and treatment options. Targeted dissemination of (intervention) research findings is thought to be a fundamental component of the scientific process,^21^ however, researchers often conclude their work once their findings are published. This results in a time lag between the publication of research results and their translation into clinical practice: Estimations of the duration of this time lag vary largely,^22^ but mostly average around 14–17 years.^23–25^ This difference between what is *known* about interventions or health problems and what is actually being *done* for health promotion and disease prevention is called the “know-do gap”.^26^ One reason for this gap is unsuccessful or insufficient dissemination (ie, communication) of scientific results: Only around 14% of scientific results are actually implemented in clinical practice, which is referred to as the “leaky pipeline”.^23^,^27^,^28^ This might partially be due to dissemination strategies that are not tailored to the desired audience. This challenge is particularly pronounced for patient populations, who often lack access to academic sources and face barriers related to complex medical terminology.^29^ Consequently, and in order to conduct successful dissemination of research results, the first step is to identify the knowledge needs of those affected by the condition, and to determine how to best reach the targeted audience.^30^

This study aimed to integrate the perspectives of adolescents with chronic pain, their caregivers, and healthcare professional to assess the current information dissemination landscape, including satisfaction, preferred sources, desired strategies, and access barriers of participants.

## Materials and Methods

This qualitative study asked three groups of interest (adolescents with chronic pain, parents, and healthcare professionals) separately about their information needs and wishes.

### Study Design

Semi-structured interviews were conducted with healthcare professionals in Switzerland who work with adolescent patients with chronic pain. To gather adolescents’ and parents’ opinions, semi-structured focus groups were conducted at the University Children’s Hospital Zurich, Switzerland. Both the interviews and the discussions were audio-recorded. The study procedure and discussion guide were approved by the Ethics Committee of the Faculty of Philosophy, University of Zurich, Switzerland (study ID: 21.12.4). All participants (ie, adolescents, parents, and healthcare professionals) received written information on the study, had the opportunity to ask questions, and provided written informed consent prior to participation. Informed consent included permission for the publication of anonymized responses and direct quotes. As the study was considered low risk, adolescents aged 15 and older were able to consent to participation by themselves.^31^ For adolescents below the age of 15, parental consent was required and obtained. The study is in compliance with the Declaration of Helsinki.^31^

### Study Participants

The study sample consisted of pediatric healthcare professionals from Switzerland, who were interviewed individually, adolescent patients with chronic pain, and their caregivers, who each participated in focus group discussions. Healthcare professionals from the member list of the Special Interest Group (SIG) Pediatric Pain from the Swiss Pain Society^32^ were contacted. Their age, professional background (medical, psychological, nursing, etc.), and years of experience were not considered as inclusion criteria. Additionally, healthcare professionals providing care for children and adolescents with chronic pain were identified through online searches using various keywords (therapy, chronic pain, children and adolescents, Switzerland).

The other samples involved adolescents aged 13 to 19 with chronic pain, as well as caregivers of children or adolescents with chronic pain. Participation was voluntary and without financial compensation. The age range of the adolescents was selected based on the assumption that adolescents in this period are capable of self-reflection and expression,^33^ and was further supported by findings from Schneider and colleagues,^34^ who reported that patients seen in the specialized pain treatment service of a large Swiss University Children’s Hospital were on average 13.95 years old, with a peak in the prevalence of chronic pain occurring between ages 13 and 20. Sufficient German language proficiency was required for participation in both focus groups to ensure participants could engage in discussions.

### Procedure

Healthcare professionals were given the option to conduct the interviews either via Zoom or in person at a location of their choice. Interviews were semi-structured, with questions exploring information needs on chronic pain in general as well as treatment options (see supplementary materials eBox 1 for the interview guideline). The interview format allowed for flexibility regarding time and place of the interview.

We conducted two focus groups: Focus Group 1 consisted of adolescents with chronic pain, while Focus Group 2 included caregivers of children or adolescents with chronic pain (see supplementary materials eBox 1 for the questions). The questions were discussed with patients and caregivers separately to avoid mutual influence, and to ensure that the specific needs of both groups were considered. Quotations from the interviews and focus groups have been translated from Swiss German to English.

### Qualitative Analysis

We applied structural content analysis based on Mayring.^35^ The first round of focus groups were coded and analyzed by two independent raters (J.H. and E.K.N)., using the online software QCAmap.www.qcamap.org.^36^ An inductive data-driven approach was applied, which enables the exploration of core themes in a phenomenon with limited existing theory or literature (see eFigure 1).^37^ A multistage analytic process was conducted.^35^ First, the transcript was worked through line by line. Text passages that aligned with the corresponding research question were coded within accordingly defined categories. After 20% of the material had been analyzed, the category system was revised in a consensus meeting with the project leader (H.K). Subsequently, the entire dataset was analyzed by the two reviewers independently, applying the identical criteria that the research group had agreed upon (ie, category definition and level of abstraction). The final list of categories was organized into main categories. Intercoder agreement was qualitatively assessed: both authors (J.H. and E.K.N.) independently reviewed the text material following all predefined content-analytical rules and discussed their coding. Remaining discrepancies were resolved in a further consensus meeting with the project leader (H.K). Finally, frequency analyses of the category occurrences in the text material were conducted.

## Results

### Sample Characteristics

Three adolescent girls (aged 14–19 years) were supposed to take part in the focus group, however one was interviewed individually, because she could not attend the focus group discussion. They reported chronic pain for 7.67 years on average. The other focus group consisted of four mothers of a child or an adolescent with chronic pain. Twelve healthcare professionals with different backgrounds were interviewed individually. See Table 1 for more information on sample characteristics.

**Table 1:** Descriptive Statistics of Samples

Adolescents	N = 3
Age, *M* (*SD)*	16.33 (2.52)
Gender, n (%)	
Female	3 (100%)
Current education level, n (%)	
Secondary School	2 (66.6%)
Apprenticeship	1 (33.3%)
Duration of chronic pain (in years), *M* (*SD)*	7.67 (3.69)
Caregivers	N = 4
Age, *M* (*range)*	48.50 (6.56)
Gender, n (%)	
Female	4 (100%)
Current job, n (%)	
Physician	1 (25%)
Teacher Gastronomy specialist	2 (50%) 1 (25%)
Healthcare professionals	N = 12
Age, *M* (*SD)*	44.7 (9.6)
Gender, n (%)	
Female	11 (91.7%)
Male	1 (8.3%)
Current job, n (%)	
Physician	4 (3.33%)
Nurse	3 (25)
Psychiatrist	2 (16.7%)
Psychologist	1 (8.3%)
Physiotherapist	1 (8.3%)
Naturopath	1 (8.3%)
Hypnotherapist	1 (8.3%)

### Dissemination of Pediatric Chronic Pain Research

In total, 843 text passages were identified and condensed into 105 categories across the three main questions (ie, where do you get your information from; how content are you with this information / what would you wish for; and what barriers do you experience while searching for information). These categories were further organized into 15 main categories and grouped into three superordinate categories (see Table 2). The coding process showed a high level of agreement between the two coders, with only minor discrepancies requiring resolution. Table 2 also depicts the group that mentioned this category (ie, adolescents, caregivers, healthcare professionals). An overview of all superordinate categories, main categories, and categories is provided in the supplement, alongside the origin of each category from the subsample and the number of quotations per category (eTable 1). The order of presentation reflects the number of quotations within each category, aligning with Mayring’s approach to complement the qualitative insights with quantitative support.^35^

**Table 2:** Superordinate Categories with Corresponding Main Categories

Superordinate Categories	Main Categories
1. Information sources used	Healthcare professionals (A, CG)
by the different groups	Digital sources (A, CG, P) Interpersonal exchange within social network (A, CG, P) Reading material (A, CG, P) Professional education (P)
2. Information needs and expectations	Preferred format of information delivery (A, P)
of different groups	Clinical and professional resource needs (P)
	Specific information content and presentation preferences (A, CG, P)
	Societal awareness of CP and education needs for social context and society (CG) Need for CP-specific expertise and support from healthcare professionals (A, CG)
3. Barriers in information search	Information related barriers (A, CG, P)
of different groups	Clinical and diagnostic barriers (A, CG, P)
	Societal awareness and education barriers (A, CG)
	Personal barriers (A, CG)
	Systemic barriers (A, CG)

**Abbreviations**: A, Adolescents; CG, Caregivers; P, Professionals.

1. Sources of information about pediatric chronic pain and its treatment: What is available?

Caregivers, adolescents, and healthcare professionals emphasized using digital sources in their search for information on pediatric chronic pain and its treatment. They mentioned informational formats such as videos and podcasts, and general online searches. Healthcare professionals specifically mentioned online scientific databases (eg, PubMed). Caregivers and adolescents mentioned a wide range of healthcare professionals as their main source of information, including pediatricians, gynecologists, psychiatrists, psychologists, physiotherapists, and chronic pain-specific pain consultation services.

Adolescent: I think the first time I heard anything about chronic pain was with my physiotherapist. And I got chronic pain quite early on. So, when I was about ten or eleven, it started to develop. My pediatric physiotherapist was actually the first to say that it looked like chronic pain, it fell into the same pattern.

Interpersonal exchanges, such as those with other adolescents with chronic pain or expert colleagues in healthcare (for the healthcare professionals) were identified by all three groups as an important source of information. Caregivers often mentioned their personal network (eg, friends and family) being of use, while adolescents specifically noted their caregivers as a significant contributor to their understanding of chronic pain and its treatment.

Adolescent: When we went to the doctors, my mother, well, she loves doing that, she asks great questions and [has the doctors] explain everything three times. And she always talked to me about it afterwards at home and explained everything to me again.

Literature, particularly scientific publications, and books was mentioned by all three groups as sources of information. Healthcare professionals specifically mentioned guidelines from specialized pain clinics in German-speaking regions and cited their own education and training as a primary source of information on chronic pain.

2. Information needs and preferences: What do patients, parents, and healthcare professionals need?

Several requests towards healthcare professionals were expressed by caregivers and adolescents. Caregivers wished for better-trained experts. Adolescents specifically desired more time during doctor visits, more information throughout the treatment phase, a more optimistic outlook from doctors, and closer medical support.

Adolescent: When you have chronic pain and it takes over your life a bit, you feel like you’ll never get out of it. You’re like, you feel like, ok this is my life now. I’m going to be in pain all my life and I’m not going to get anything done. And then maybe I would have wished that people had shown more hope and instilled more hope.

Adolescents expressed the need for information to be presented to them in a clear and concise way. They indicated that summaries in various formats – such as flyers, brochures, websites, and videos – would be most beneficial. It became evident that searching for information independently often poses challenges for adolescents, and therefore, a well-organized presentation of the information would be highly sought after.

Adolescent: But also, something like an overview sheet, where you get an overview of what has been discussed [during the medical appointment] and simply what it [chronic pain] is and how to handle it. Something like that is what I would have wished for.

Caregivers expressed their desire for greater acceptance and understanding of chronic pain within the broader society. They believed that society has not yet fully recognized chronic pain as a legitimate medical condition of its own, especially in children and adolescents, resulting in negative consequences for their children, such as lack of understanding of their limitations in daily life.

Caregiver: And that’s still a bit of a pain when you’re 16, and everyone goes out and the bus doesn’t run in the evening [....]. And the boys would love to accompany my daughter home by bike. [They tell her] ‘Hey, are you stupid, you can’t ride a bike or what?’ But she can’t say ‘Hey, I have chronic pain, that’s why I can’t ride a bike. I never learned how to do it [ride a bike].’

Adolescents also expressed specific wishes regarding the information, such as it being tailored to the individual patient and coming from trusted and verified sources. Both adolescents and caregivers expressed their wish for information to be presented in a simple and comprehensive manner. Adolescents also mentioned that they did not want to be overwhelmed with information.

Adolescent: if I see that the video is 30 minutes long, then I certainly won’t watch it (laughs).

Finally, healthcare professionals emphasized their need for Switzerland-specific data, more studies, treatment-specific guidelines, improved therapy options, further education and training, and a platform for interdisciplinary exchange.

Healthcare professional: I would like to see more [treatment-related] guidelines for children and adolescents in particular. In this particular area, studies are limited to independent [case] reports and expert opinions at best, and I would like to see more evidence-based studies that can then be summarized in guidelines.

3. Barriers in information gathering: What challenges exist?

Adolescents and caregivers reported various personal barriers, such as exhaustion, feelings of powerlessness, and guilt or self-doubt. Some adolescents reported suppressing thoughts of their condition due to fluctuating symptoms, periods of relief leading to avoidance of confronting the issue, and hope that the problem might resolve itself without intervention. They described their belief that reading about their condition would not contribute to their recovery and was therefore pointless.

Adolescent: Because sometimes I get the feeling that there is nothing left for me to do, nothing that could help.

Various societal barriers were emphasized. Caregivers mentioned the frequent unsolicited advice they receive from acquaintances. Further, caregivers and adolescents felt that healthcare professionals did not take them seriously and expressed a sense of being misunderstood by their families and society in general.

Adolescent: [The doctor who diagnosed me in Germany], he was the first person to take me seriously. He really believed that I wasn’t just some kind of “sensitive little thing” that pretends [to be in pain].

The participants identified several organizational challenges, including limited time of healthcare professionals, high monetary costs associated with diagnostic procedures or treatments that are not always covered by health insurance, and difficulties accessing experts and making appointments. They mentioned experiences with physicians who lacked a holistic view of the patient and instead focused only on finding organic causes of the pain. Healthcare professionals pointed out the complexity of chronic pain as a barrier. Finally, caregivers noted a lack of knowledge among experts and the challenges associated with the absence of physical or psychological diagnoses or contradictory diagnoses.

Caregiver: I am fed up with specialists who earn so much [money], and then I, as a mother with no medical training, have to explain to them what chronic pain is. (Sighs) They have no empathy towards me. They make me feel angry and aggressive.

Lastly, caregivers and adolescents discussed several specific information-related barriers, such as a lack of accessible and free resources, unreliable or unverified sources, an overwhelming quantity of information, and the fact that the information is not tailored to the individual patient. Healthcare professionals also emphasized the information gap between research on children and adolescents with chronic pain compared to adults, along with the overall scarcity of information and the limited data available for Switzerland.

## Discussion

The current study aimed to investigate information sources on pediatric chronic pain actually used by adolescents with chronic pain, caregivers, and healthcare professionals; their preferences on how they would like to receive this information, and barriers they experienced when looking for information. Our findings indicate that while there were areas of overlap, each group also expressed specific needs and expectations. Adolescents and caregivers reported encountering societal, clinical, systemic, and information-related challenges when seeking information. Healthcare professionals primarily faced clinical and information-related challenges, and specifically emphasized the limited availability of relevant research (ie, in pediatric populations and with a focus on Switzerland). The study’s approach of involving key groups in the dissemination of research aligns with the principles of *integrated knowledge translation*.^38^ This model emphasizes the active involvement of knowledge users across all stages of the research process rather than engaging them only in the final phase. Such sustained collaboration fosters mutual understanding and shared ownership of the outcomes. Moreover, it enhances the relevance, uptake, and practical application of research findings, thereby helping to close the gap between evidence generation and implementation.^27^,^38^

In our study, adolescents and caregivers emphasized the abundance of information available but noted the lack of access to verified online sources, as these are often behind paywalls or only accessible within academic networks. In contrast, healthcare professionals described greater accessibility to evidence-based online resources, which is consistent with prior findings.^39^ These observations highlight the importance of directing patients and their families toward existing curated, trustworthy platforms and online initiatives such as *#itdoesnthavetohurt* (itdoesnthavetohurt.ca), an English-language evidence-based communication campaign led by Professor Christine Chambers at Dalhousie University, which aims to improve the dissemination of evidence-based information on pediatric pain.

For adolescents and caregivers, most information was obtained through interpersonal exchange, primarily with healthcare professionals. However, both groups frequently reported encountering a lack of knowledge among various healthcare professionals and, following their own research, often perceived themselves as more informed about chronic pain than the experts they consulted. This knowledge gap may be attributed to the limited training many healthcare professionals receive in pediatric chronic pain,^13^,^15^,^16^,^19^ resulting in a predominantly one-dimensional explanation of chronic pain – such as a purely biomedical or psychological perspective – used by healthcare professionals during pain education.^14^,^19^,^40^,^41^ Effective and structured pain education, and a clear and empathic communication style to foster good patient-healthcare professional relationships can help ensure a more comprehensive biopsychosocial understanding of patients and their families.^42–44^

Healthcare professionals reported relying on interpersonal exchange within interdisciplinary, national and international networks, and scientific sources. A notable concern was the considerable research gap between studies focused on pediatric versus adult populations. Some Swiss prevalence rates of pediatric chronic pain are available, such as the 3.62% prevalence estimation based on pediatricians’ assessments in medical practices,^15^ which is in sharp contrast to the up to 20% prevalence rates based on adolescents’ self-reports.^45^ Hence, comprehensive and precise epidemiological data remain insufficient. Healthcare professionals unanimously emphasized the importance of consolidating relevant information in a centralized resource, either in analog form or through digital repositories and platforms, which is also recommended in clinical guidance literature.^46^

Healthcare professionals predominantly focused on scientific content, education, and professional exchange in relation to pediatric chronic pain and its treatment. While an evidence-based knowledge base was crucial for all participant groups, adolescents and caregivers faced challenges that go beyond content alone. They emphasized the need for addressing healthcare system-related issues, emotional, and societal factors. This discrepancy highlights the importance of engaging diverse stakeholder groups to not only ensure a comprehensive understanding of the complex challenges associated with pediatric chronic pain, but also to actively support the development and implementation of targeted solutions for different groups. Facilitating such engagement can be supported through established networks, such as Solutions for Kids in Pain (SKIP), which connect stakeholders and foster collaborative knowledge mobilization.^47^ However, as SKIP is a Canada-based initiative available in French and English and shaped by the specific features of the Canadian healthcare system, its applicability to other contexts may be limited and would require adaptation to local structures and needs.

Contextual factors can also affect patients and should therefore be considered in pain assessment protocols and educational efforts. For example, participants expressed a desire for exchange between individuals with chronic pain. Such peer interactions can have a relieving and hope-inducing effect.^48^ Within the family context, caregivers awareness of their role in navigating their child’s pain can also be influential.^49^,^50^ Even more broadly, adolescents and caregivers considered societal awareness to be crucial. A fundamental concern of patients and families, underscored by findings from Koesling et al (2019),^51^ was the recognition that chronic pain is real, does indeed also affect children and adolescents, and needs to be taken seriously. Relatedly, an emerging and growing area of research focuses on experiences of pain-related stigma.^52^ Pain-related stigma can take various forms and originate from peers, school staff, healthcare professionals and family members, significantly impacting the well-being of individuals with chronic pain.^53–56^

## Strengths and Limitations

To the best of our knowledge, this is the first study to examine information sources currently used, information-specific desires and barriers for different groups affected by pediatric chronic pain in Switzerland. The qualitative approach facilitated an in-depth exchange and understanding of families’ and healthcare professionals’ experiences and needs. This provides a comprehensive view of the information landscape on pediatric chronic pain in Switzerland and lays the foundation for further initiatives to improve access to evidence-based, specific and tailored information for all those affected by pediatric chronic pain.

Despite these strengths, our study also comes with several limitations: First, families were recruited from a single University children’s hospital in Switzerland, which limits the external validity of the results. Second, the sample lacked sufficient diversity, particularly in terms of gender, race, and socio-economic status, which may affect the generalizability of the findings across different demographic groups. The overrepresentation of individuals with high socioeconomic status is commonly observed,^57^ and suggests a pattern of socioeconomic disparities in research participation. Third, the sample exhibited a marked gender imbalance across all participant groups. Only one healthcare professional was male, while all of the adolescents and caregivers identified as female. This imbalance may have influenced the perspectives represented in the data, as experiences of chronic pain and related information needs may differ by gender. Consequently, the transferability of the findings to male adolescents, caregivers, and healthcare professionals may be limited. Future studies should aim for more balanced gender representation to better capture potential gender-related differences in information needs and experiences. Fourth, individual pain duration for participants with chronic pain was not retained due to a data processing error; only aggregated data (mean, SD) were available. This limits the examination of potential relationships between individual pain duration and information needs or preferences. Fifth, data on the healthcare professionals’ length of experience were not systematically collected, which limits the assessment of its influence on perspectives regarding information needs and practices in pediatric chronic pain. Future research should include this variable to better understand how professional experience shapes information-related practices and priorities. Sixth, healthcare professionals were recruited from the Special Interest Group on Pediatric Pain, Swiss Pain Society’s member list, and were thus already rather familiar with the topic. Including healthcare professionals with less or no experience in pediatric chronic pain might have enabled a more extensive understanding of the information situation of those who do not work with adolescents with chronic pain on a daily basis.

## Conclusion and Future Directions

Our analysis identified key themes, including information-related, organizational and personal barriers in the search for information, and needs related to societal understanding and the healthcare system in general. The findings indicate that dissemination of scientific results on pediatric chronic pain remains inadequate and highlight ways to improve it. This study paves the way for more specific and targeted dissemination of scientific findings, ensuring that patients, families, and other stakeholders can effectively access relevant information to make informed decisions.

Future research should prioritize the creation of a centralized platform for verified, recipient-specific information, as well as the promotion of interdisciplinary professional exchanges. Additionally, there is a clear need for the development of targeted, interdisciplinary training programs for healthcare professionals to address the existing gaps in knowledge about the diagnosis and treatment of pediatric chronic pain. Finally, incorporating co-design methodologies in the development and refinement of dissemination strategies would help ensure that solutions are better tailored to the needs of both patients and professionals.

## Data Availability

Data generated in this study is available upon request directed towards the corresponding author, Helen Koechlin, PhD.
